# MEK1/2 Inhibitor (U0126) and PI3K Inhibitor (LY294002) Suppress Herpes Simplex Virus Type 1 Replication by Targeting MAPK/ERK1/2 and PI3K/AKT Signaling Pathways: Implications for Oral Health and Translational Control of Orolabial HSV-1 Infection

**DOI:** 10.5812/ijpr-164639

**Published:** 2026-01-04

**Authors:** Wei Meng, Zahra Zahid Piracha, Umar Saeed, Dilber Uzun Ozsahin, Ilker Ozsahin, Yongwei Tao, Zhanping Ren

**Affiliations:** 1 Oral and Maxillofacial Surgery, Hulunbuir People’s Hospital, Hulunbuir, China; 2 Szechenyi Istvan University, Gyor, Hungary; 3 Clinical and Biomedical Research Center (CBRC), Foundation University School of Health Sciences (FUSH), Foundation University Islamabad (FUI), Islamabad, Pakistan; 4 Operational Research Center in Healthcare, Near East University, Mersin, Cyprus, Turkey; 5 University College, Korea University, Seoul 02418, Republic of Korea (South Korea); 6 International Center of Medical Sciences Research (ICMSR), Islamabad, Pakistan; 7 International Center of Medical Sciences Research (ICMSR), Essex, United Kingdom; 8 International Center of Medical Sciences Research (ICMSR), Austin, TX, United States of America; 9 Medical Department of Diagnostic Imaging, College of Health Sciences, University of Sharjah, Sharjah, United Arab Emirates; 10 Key Laboratory of Shaanxi Province for Craniofacial Precision Medicine Research, College of Stomatology, Xi'an Jiaotong University, Xi’an, China; 11 Laboratory Center of Stomatology, College of Stomatology, Xi'an Jiaotong University, Xi'an, China; 12 Department of Cleft Palate-Craniofacial Surgery, College of Stomatology, Xi'an Jiaotong University, Xi'an, China

**Keywords:** Keywords:, U0126, LY294002, HSV-1, ERK1/2 Signaling, PI3K/AKT Pathway, HaCaT Cells, Keratinocytes, Viral Replication, Inflammation, Host-Directed Therapy

## Abstract

**Background:**

Current antivirals for orolabial Herpes simplex virus type 1 (HSV-1) often provide incomplete suppression and limited reactivation control, sustaining recurrent oral lesions and inflammation that compromise oral health. HSV-1 subverts host signaling networks to enhance its replication and trigger inflammation. Among these, the extracellular signal-regulated kinase 1/2 (ERK1/2) and phosphatidylinositol 3-kinase/protein kinase B (PI3K/AKT) pathways are hijacked to facilitate viral gene expression and cell survival.

**Objectives:**

In this study, we employed U0126 [a mitogen-activated protein kinase 1/2 (MEK1/2) inhibitor] and LY294002 [a phosphatidylinositol 3-kinase (PI3K) inhibitor] as targeted pharmacological tools to intercept HSV-1’s exploitation of host keratinocyte signaling.

**Methods:**

Human HaCaT keratinocytes were infected with HSV-1 and treated with U0126 or LY294002. Western blotting was used to assess phosphorylation of ERK1/2 and activation of protein kinase B (AKT). MTT assays were performed to evaluate cell viability. Real-time PCR was utilized to quantify viral transcripts (ICP0, ICP4, gB, and gC) and inflammatory cytokines [interleukin-6 (IL-6) and tumor necrosis factor-alpha (TNF-α)]. Confocal microscopy was employed to visualize the intracellular distribution of phosphorylated extracellular signal-regulated kinase 1/2 (p-ERK1/2), phosphorylated activation of protein kinase B (p-AKT), and HSV-1 glycoprotein D (gD). Viral titers were determined using plaque assays.

**Results:**

The HSV-1 infection induced a time-dependent increase in phosphorylation of ERK1/2 and AKT, with p-ERK1/2 peaking at 12 h and p-AKT increasing 2.5-fold by 24 h. Cell viability declined from 100% at baseline to 45% at 24-hours post-infection (hpi). Treatment with U0126 and LY294002 reduced p-ERK1/2 and p-AKT levels to 25% and 30% of infected controls, respectively, restoring viability to 82 - 86%. Both inhibitors markedly suppressed viral gene expression (ICP0, ICP4, gB, gC down by 60 - 80%) and inflammatory cytokines (IL-6 and TNF-α reduced by > 50%). Plaque assays showed a strong decline in infectious titers — from 175 plaques per well in untreated infection to 60 and 45 plaques after U0126 and LY294002, respectively. Confocal imaging further revealed diminished nuclear accumulation of p-ERK1/2 and p-AKT, indicating disruption of post-entry signaling critical for viral replication.

**Conclusions:**

Targeting host signaling bottlenecks with U0126 and LY294002 offers a dual-pronged antiviral strategy against HSV-1 by dismantling the ERK/AKT axis critical for replication and inflammatory amplification. These findings position MEK1/2 and PI3K as promising therapeutic nodes for managing cutaneous HSV-1 infections. This host-directed dual-pathway inhibition may therefore help reduce recurrent orolabial HSV-1 lesions.

## 1. Background

Oral health is undermined by the cyclical reactivation of Herpes simplex virus type 1 (HSV-1) at the orolabial mucosa, where recurring lesions and inflammatory bursts persist despite conventional antiviral control. HSV-1 is a widespread human pathogen responsible for a range of clinical conditions, from mild orofacial lesions to severe complications such as encephalitis and keratitis, which may involve the nervous system and ocular tissues (1, 2). As a double-stranded DNA virus of the *Herpesviridae* family, HSV-1 exhibits a complex replication cycle and establishes lifelong latency within sensory neurons (3). Reactivation of the latent virus under stress or immunosuppression results in recurrent infections, making HSV-1 a persistent public health concern worldwide (4-6). Although antiviral drugs such as acyclovir remain the mainstay of therapy, rising drug resistance and the inability to eliminate latent viral reservoirs underscore the need for new therapeutic strategies that interfere with host–virus interactions at the molecular level (7, 8).

Upon entering epithelial cells, HSV-1 activates multiple intracellular signaling cascades that alter host cellular functions to favor viral replication, immune evasion, and pathogenesis (9). Among these, the mitogen-activated protein kinase (MAPK) and phosphatidylinositol 3-kinase/protein kinase B (PI3K/AKT) pathways are of particular interest, as they regulate cell proliferation, survival, and inflammatory responses. Previous studies have shown that HSV-1 exploits extracellular signal-regulated kinase 1/2 (ERK1/2) signaling to promote immediate-early gene transcription and drive productive infection (10, 11). Likewise, activation of the PI3K/AKT pathway enhances viral genome replication, suppresses apoptosis, and modulates antiviral immune responses, creating a cellular environment conducive to viral persistence (12, 13). However, the timing, interplay, and relative contributions of these pathways during HSV-1 infection in keratinocytes remain insufficiently understood.

Keratinocytes ,especially those represented by the HaCaT cell line, are physiologically relevant to HSV-1 pathogenesis because they form the outermost epithelial barrier at mucocutaneous sites of viral entry and reactivation (14, 15). HaCaT cells are spontaneously immortalized yet non-tumorigenic human keratinocytes that retain differentiation capacity and robust innate immune signaling, making them an ideal in vitro model for studying virus–host interactions in the skin and mucosa.

To specifically investigate the functional roles of these signaling pathways, we employed two well-established pharmacological inhibitors: U0126 and LY294002. U0126 is a potent and selective inhibitor of mitogen-activated protein kinase 1/2 (MEK1/2) that blocks downstream phosphorylation of ERK1/2, whereas LY294002 is a classical PI3K inhibitor that prevents activation of protein kinase B (AKT) by competing at the ATP-binding site. Both compounds are widely used in molecular virology and cancer biology to delineate pathway-specific mechanisms. Importantly, U0126 and LY294002 possess well-established safety and specificity profiles in cell-based models, allowing selective inhibition of the MAPK/ERK and PI3K/AKT axes without inducing nonspecific cytotoxicity.

## 2. Objectives

Their use in this study provided a mechanistic approach to determine whether HSV-1 replication and inflammatory responses depend on these host signaling cascades, thereby identifying potential host-directed antiviral targets that could complement conventional therapeutic strategies.

## 3. Methods

### 3.1. Cell Culture and Reagents

HaCaT keratinocytes (immortalized, non-tumorigenic human epidermal cells widely accepted as a standard in vitro model for studying epithelial viral infections) were cultured as defined earlier (13) in Dulbecco’s Modified Eagle Medium (DMEM; Gibco) supplemented with 10% fetal bovine serum (FBS; Gibco), 1% penicillin-streptomycin (100 U/mL and 100 µg/mL, respectively), and 1% L-glutamine. The selection of HaCaT cells was based on their stable differentiation capacity, intact innate immune signaling, and proven susceptibility to HSV-1 infection without requiring viral adaptation. Cells were maintained at 37°C in a humidified atmosphere containing 5% CO_2_. The HSV-1 (strain F) was propagated in Vero cells and titrated using plaque assays. For all infection experiments, HaCaT cells were infected at a multiplicity of infection (MOI) of 1.0.

Inhibitors:

- U0126 (MEK1/2 inhibitor, Sigma-Aldrich) was dissolved in 100% DMSO to prepare a 10 mM stock solution, which was further diluted in culture medium to achieve a final working concentration of 10 µM.

- LY294002 (PI3K inhibitor; Sigma-Aldrich) was dissolved in DMSO to obtain a 20 mM stock and used at a final concentration of 20 µM.

- Cells were first treated with inhibitors for an hour before the virus was introduced and kept in this medium for the entire experiment (14).

### 3.2. Herpes Simplex Virus Type 1 Infection and Time-Course Sampling

HaCaT cells were plated in 6-well plates at 2 × 10^5^ cells/well or in 24-well plates at 5 × 10^4^ cells/well, reaching approximately 70% confluency at the time of infection. Cells were exposed to HSV-1 (MOI = 1) in 500 µL serum-free medium per well for 1 hour at 37°C with gentle rocking every 15 minutes. After adsorption, the inoculum was removed, and cells were washed twice with 1 mL sterile PBS. Fresh complete medium (2 mL per well for 6-well plates; 1 mL per well for 24-well plates) with or without inhibitors was added. Samples were harvested at 0-, 6-, 12-, and 24-hours post-infection (hpi) for protein, RNA, and cell viability analyses.

### 3.3. Western Blotting

Cells were lysed in 200 µL RIPA buffer containing protease and phosphatase inhibitors (Thermo Fisher) per well of a 6-well plate. Protein concentrations were determined using the BCA assay. Thirty micrograms (30 µg) of total protein were separated on 10% SDS-PAGE gels and transferred onto PVDF membranes (0.45 µm; Millipore). All primary antibodies (Cell Signaling Technology) were used at 1:1000 dilution, and HRP-conjugated secondary antibodies at 1:5000 dilution. Membranes were developed using ECL reagents (Thermo Fisher), and densitometric quantification was performed using ImageJ software, normalized to total protein.

### 3.4. Cell Viability (MTT) Assay

The MTT assay was performed to evaluate the cytotoxic effects of HSV-1 infection and to determine whether pharmacological inhibition of the ERK1/2 or PI3K/AKT pathways could restore cell viability. HaCaT cells were seeded in 96-well plates at 1 × 10^4^ cells/well in 100 µL medium and allowed to attach overnight. Cells were pretreated with U0126 (10 µM) or LY294002 (20 µM) for 1 h before HSV-1 infection (MOI = 1) and maintained in inhibitor-containing medium for the duration of the experiment. Viability was assessed at 0, 6, 12, and 24 hpi to monitor progressive cytopathic effects over time. These time points were selected to represent early (viral entry), mid (gene expression), and late (replication) phases of the HSV-1 life cycle. At each time point, 10 µL of MTT solution (5 mg/mL stock; final 0.5 mg/mL) was added and incubated for 4 h at 37°C. The medium was then replaced with 100 µL DMSO to dissolve formazan crystals, and absorbance was measured at 570 nm using a BioTek microplate reader. Preliminary dose-response tests confirmed that 10 µM U0126 and 20 µM LY294002 were nontoxic to HaCaT cells (> 90% viability), and therefore IC_50_ values were not specifically determined, as both inhibitors were used at well-established sub-cytotoxic concentrations reported in prior studies (14).

### 3.5. Quantitative Real-time PCR

Total RNA was extracted using the TRIzol reagent (Invitrogen) according to the manufacturer’s protocol. RNA (1 µg) was reverse-transcribed using the RevertAid First Strand cDNA Synthesis Kit (Thermo Fisher) in a 20 µL reaction volume. The qRT-PCR was carried out in 20 µL reactions containing 10 µL SYBR Green Master Mix (Applied Biosystems), 0.5 µM of each primer, and 2 µL of cDNA template. Gene-specific primers targeted HSV-1 immediate-early (ICP0, ICP4), late (gB, gC) genes, and host cytokines [interleukin-6 (IL-6) and tumor necrosis factor-alpha (TNF-α)]. Relative expression levels were calculated using the 2^-ΔΔCt^ method, normalized to GAPDH (16, 17). The primer sequences used for qRT-PCR are listed in Table 1. 

**Table 1.** Primer Sequences Used for Quantitative Real-time PCR

Genes and Primer Type	Sequence (5′→3′)	Amplicon Size (bp)
**HSV-1 ICP0**		143
Forward	CGCTGTTCTCCTTGGACTTG	
Reverse	TTGTTGGGCGTGTTTCTTGT	
**HSV-1 ICP4**		175
Forward	GCTCCGAAGAACTGGACTGA	
Reverse	CTTGGTGACGTGGTTGTTGT	
**HSV-1 gB**		118
Forward	GGACGAGGCGCCGTTTACGA	
Reverse	AGCAGGGTGCTCGTGTTTGG	
**HSV-1 gC**		132
Forward	CTTGACGACGATGACGACGA	
Reverse	GTTGTCGTCGTCGTAGTCGT	
**IL-6 (human)**		149
Forward	ACTCACCTCTTCAGAACGAATTG	
Reverse	CCATCTTTGGAAGGTTCAGGTTG	
**TNF-α (human)**		136
Forward	CCTCTCTCTAATCAGCCCTCTG	
Reverse	GAGGACCTGGGAGTAGATGAG	
**GAPDH (human, control)**		121
Forward	GAAGGTGAAGGTCGGAGTC	
Reverse	GAAGATGGTGATGGGATTTC	

Abbreviations: HSV-1, Herpes simplex virus type 1; TNF-α, tumor necrosis factor-alpha; IL-6, interleukin-6.

### 3.6. Immunofluorescence and Confocal Microscopy

HaCaT cells were grown on sterile glass coverslips (12 mm diameter; 1 × 10^5^ cells/coverslip) in 24-well plates. After treatment, cells were fixed with 4% paraformaldehyde for 15 min, permeabilized with 0.1% Triton X-100 for 10 min, and blocked in 1% BSA for 30 min. Primary antibodies against phosphorylated extracellular signal-regulated kinase 1/2 (p-ERK1/2), phosphorylated activation of protein kinase B (p-AKT), and HSV-1 glycoprotein D (gD, 1:100 dilution) were applied overnight at 4°C. Alexa Fluor–conjugated secondary antibodies (Invitrogen, 1:500 dilution) were added for 1 hour at room temperature, followed by DAPI staining (1 µg/mL, 5 min). Coverslips were mounted with antifade reagent and imaged using a Leica SP8 confocal microscope.

### 3.7. Plaque Assay

Viral titers were determined using standard plaque assays in Vero cells (2 × 10^5^ cells/well in 6-well plates). Cell-free supernatants collected 24 hpi were serially diluted 10-fold (10^-1^ to 10^-6^) in serum-free medium. 500 µL of each dilution was added to Vero monolayers and incubated for 1 hour at 37°C with gentle rocking. After adsorption, the inoculum was replaced with 2 mL of DMEM containing 2% FBS and 0.8% methylcellulose. Plates were incubated for 72 hours, fixed with 4% paraformaldehyde for 20 min, and stained with 0.5% crystal violet. Plaques were counted, and titers were expressed as plaque-forming units per milliliter (PFU/mL).

### 3.8. Enzyme-Linked Immunosorbent Assay

Levels of IL-6 and TNF-α in cell culture supernatants were quantified using human DuoSet ELISA kits (R&D Systems, Minneapolis, MN, USA) according to the manufacturer’s instructions. Briefly, 96-well plates were coated overnight with capture antibodies, blocked with 1% BSA in PBS, and incubated with standards and samples (100 µL/well) for 2 h at room temperature. After washing, biotinylated detection antibodies and streptavidin-HRP were added sequentially, followed by substrate (TMB). The reaction was stopped with 2 N H_2_SO_4_, and absorbance was measured at 450 nm using a BioTek microplate reader. Cytokine concentrations were calculated from standard curves and expressed as pg/mL.

### 3.9. Statistical Analysis

All experiments were conducted in biological triplicates (n = 3), and data are presented as mean ± standard deviation (SD). Statistical comparisons were performed using one-way ANOVA followed by Tukey’s post-hoc test in GraphPad Prism 9, with P < 0.05 considered statistically significant.

## 4. Results

### 4.1. Herpes Simplex Virus Type 1 Infection Induces extracellular Signal-Regulated Kinase 1/2 and Activation of Protein Kinase B with a Concurrent Decline in HaCaT Cell Viability

**Figure 1.**
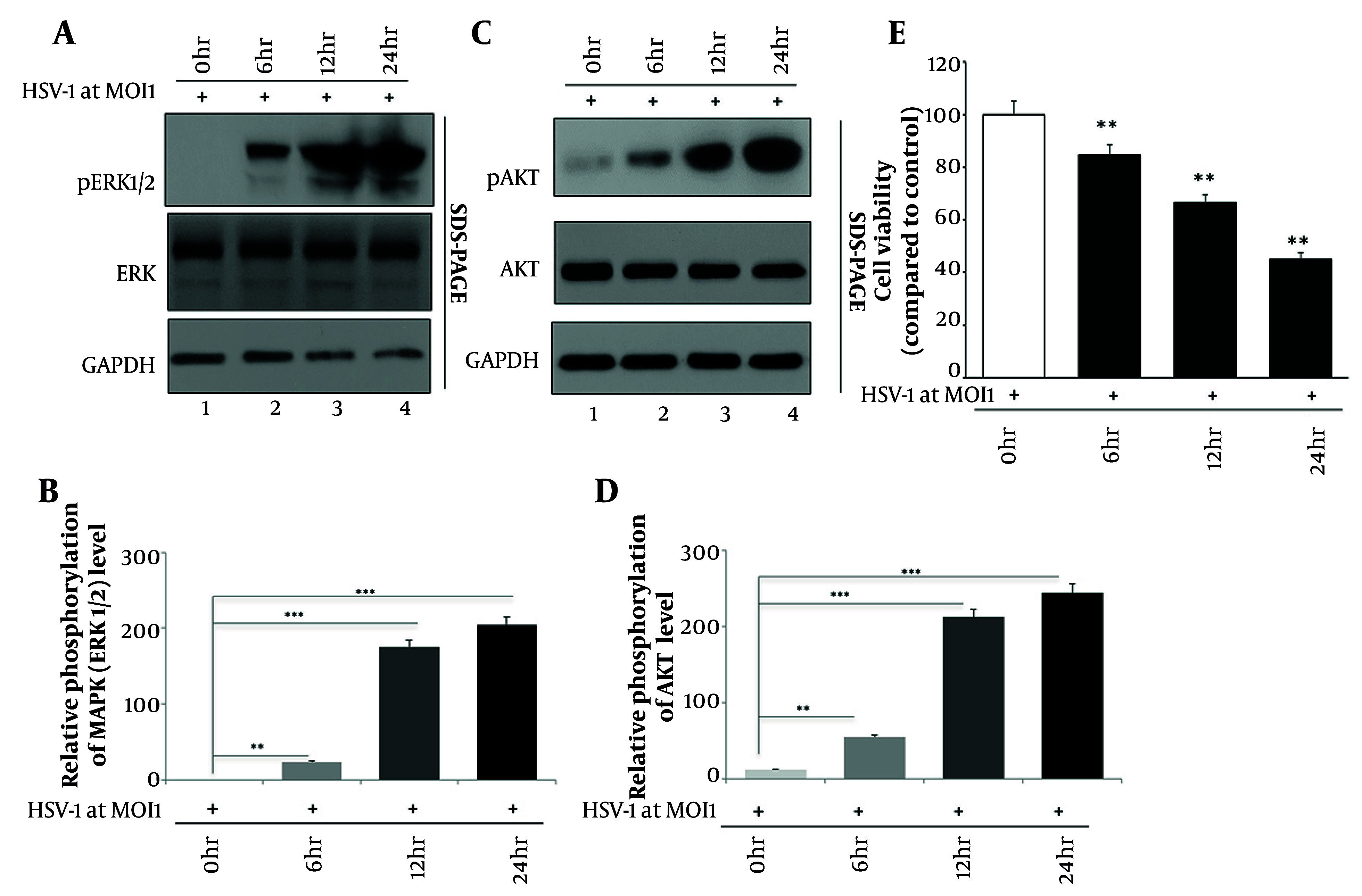
Herpes simplex virus type 1 (HSV-1) infection activates extracellular signal-regulated kinase 1/2 (ERK1/2) and activation of protein kinase B (AKT) signaling and decreases cell viability in HaCaT cells: A, Western blot analysis showing time-dependent phosphorylation of ERK1/2 following HSV-1 infection [multiplicity of infection (MOI) = 1]; B, densitometric quantification of phosphorylated extracellular signal-regulated kinase 1/2 (p-ERK1/2) normalized to total ERK1/2; C, Western blot showing phosphorylation of AKT at corresponding time points; D, densitometric quantification of phosphorylated activation of protein kinase B (p-AKT) normalized to total AKT; E, MTT assay showing progressive decline in cell viability from 0 to 24-hours post-infection (hpi). GAPDH served as the loading control [data are expressed as mean ± standard deviation (SD); n = 3; ** P < 0.01 and *** P < 0.001 vs. control].

The MAPK pathway, including ERK1/2 phosphorylation, is known to regulate various cellular processes such as proliferation, differentiation, and immune responses. Understanding how HSV-1 infection modulates this pathway can provide insights into the molecular mechanisms underlying viral pathogenesis and host-cell interactions. We performed Western blot analysis to demonstrate that HSV-1 infection led to a time-dependent increase in the phosphorylation of ERK1/2 in HaCaT cells (Figure 1A). Phosphorylation levels were measured at 0-, 6-, 12-, and 24-hpi. The results showed a time-dependent increase in ERK1/2 phosphorylation, which rose markedly by 12 hours and reached its maximum at 24-hpi, indicating sustained activation of the MAPK pathway in response to HSV-1 infection. The prolonged elevation of ERK1/2 phosphorylation up to 24 hours suggests that both early and intermediate signaling phases contribute to the host cell’s response to HSV-1 replication. This prolonged activation could be essential for effective antiviral responses and may contribute to the cellular environment necessary for viral replication and spread. Densitometric analysis relative to total ERK1/2 confirmed this trend, showing a significant elevation in p-ERK1/2 at 24-hpi (Figure 1B). 

Activation of the PI3K/AKT pathway in response to HSV-1 infection plays a role in regulating cell survival, proliferation, and metabolism. Activation of AKT signaling is critical for various cellular functions and can be hijacked by viruses to promote their replication and survival within host cells. Understanding the dynamics of PI3K/AKT pathway activation during HSV-1 infection can provide insights into viral pathogenesis and potentially identify targets for therapeutic intervention. Upon HSV-1 infection, HaCaT cells exhibited increased phosphorylation of AKT, as detected by Western blot analysis (Figure 1C). Phosphorylation of AKT was evident as early as 6-hpi, with further activation observed at 12 and 24 hours. These findings suggest a rapid and sustained activation of the PI3K/AKT pathway in response to HSV-1 infection in HaCaT cells. The early detection of AKT phosphorylation at 6-hpi, with sustained activation observed up to 24 hours, highlights the importance of both early and intermediate responses in the cellular reaction to HSV-1 infection. Quantification revealed approximately a 2.5-fold rise in p-AKT levels by 24 hours, compared to baseline (Figure 1D), indicating sustained pathway activation. This rapid activation might be crucial for initiating survival and proliferative signals that help the host cell cope with viral stress, while the sustained activation could support ongoing viral replication and persistence within the host.

To evaluate the functional impact on cellular viability, we performed MTT assays at the same time points. The HSV-1 infection resulted in a gradual reduction in viability, from 100% at baseline to 84% at 6 hours, 66% at 12 hours, and 45% at 24-hpi (Figure 1E). These findings suggest that while HSV-1 activates both ERK and AKT signaling cascades, this activation is accompanied by a progressive loss in metabolic activity, reflecting a potential shift toward viral cytopathogenicity or host stress responses. In all experiments, U0126 and LY294002 were added 1 hour prior to viral infection and maintained in the culture medium for the full 24-hour infection period to ensure continuous inhibition of the respective signaling pathways. Statistical significance in all viability assays was determined relative to both uninfected and HSV-1-infected controls (** P < 0.01, *** P < 0.001).

### 4.2. Upregulation of Viral and Host Inflammatory Genes in Response to Herpes Simplex Virus Type 1 Infection

**Figure 2.**
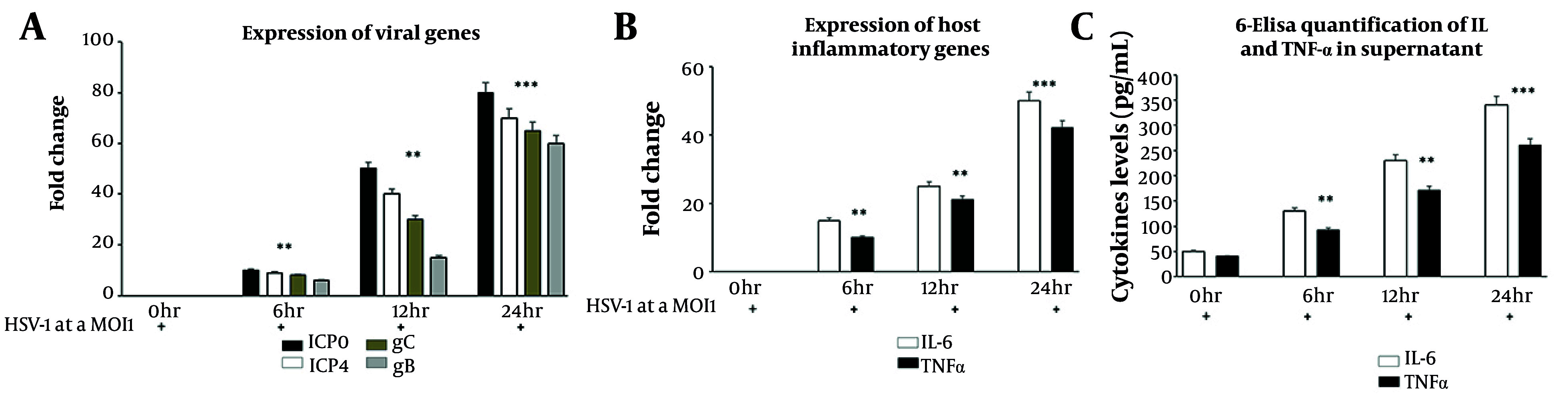
Herpes simplex virus type 1 (HSV-1) infection upregulates viral and inflammatory gene expression in HaCaT keratinocytes: A, qRT-PCR analysis showing time-dependent increases in viral transcripts (ICP0, ICP4, gB, and gC); B, qRT-PCR of host cytokines [interleukin-6 (IL-6) and tumor necrosis factor-alpha (TNF-α)] demonstrating parallel induction of inflammatory genes; C, enzyme-linked immunosorbent assay (ELISA) confirming increased secretion of IL-6 and TNF-α in culture supernatants. Results are normalized to GAPDH [data are expressed as mean ± standard deviation (SD); n = 3; ** P < 0.01, *** P < 0.001 vs. control].

To investigate the transcriptional response of HaCaT cells to HSV-1 infection, we evaluated the temporal expression of viral and host genes by real-time PCR. Viral immediate-early genes (ICP0, ICP4) and late structural genes (gB, gC) were significantly upregulated in a time-dependent manner across 0-, 6-, 12-, and 24-hpi (Figure 2A). Immediate-early genes, essential for initiating viral transcription, showed marked induction as early as 6 hours, while late genes involved in viral assembly and replication showed progressive increases toward 24 hours. These results reflect a coordinated activation of the HSV-1 gene expression program during infection. In parallel, host inflammatory genes IL-6 and TNF-α were also significantly induced in response to infection (Figure 2B). Both cytokines displayed a rapid and sustained transcriptional upsurge, suggesting an early activation of pro-inflammatory signaling pathways as part of the innate immune response. To validate whether this transcriptional induction translated into functional cytokine release, enzyme-linked immunosorbent assay (ELISA) assays were performed on culture supernatants. Protein-level analysis confirmed a corresponding time-dependent increase in secreted IL-6 and TNF-α, supporting the functional relevance of gene expression changes (Figure 2C). The combined gene expression profile illustrates the synchronized upregulation of viral and inflammatory pathways, potentially contributing to both viral propagation and host defense mechanisms.

### 4.3. Pharmacological Inhibition of Mitogen-Activated Protein Kinase and Phosphatidylinositol 3-Kinase/Protein Kinase B Pathways Attenuates Signaling Activation and Restores Cell Viability in Herpes Simplex Virus Type 1-Infected HaCaT Cells

**Figure 3.**
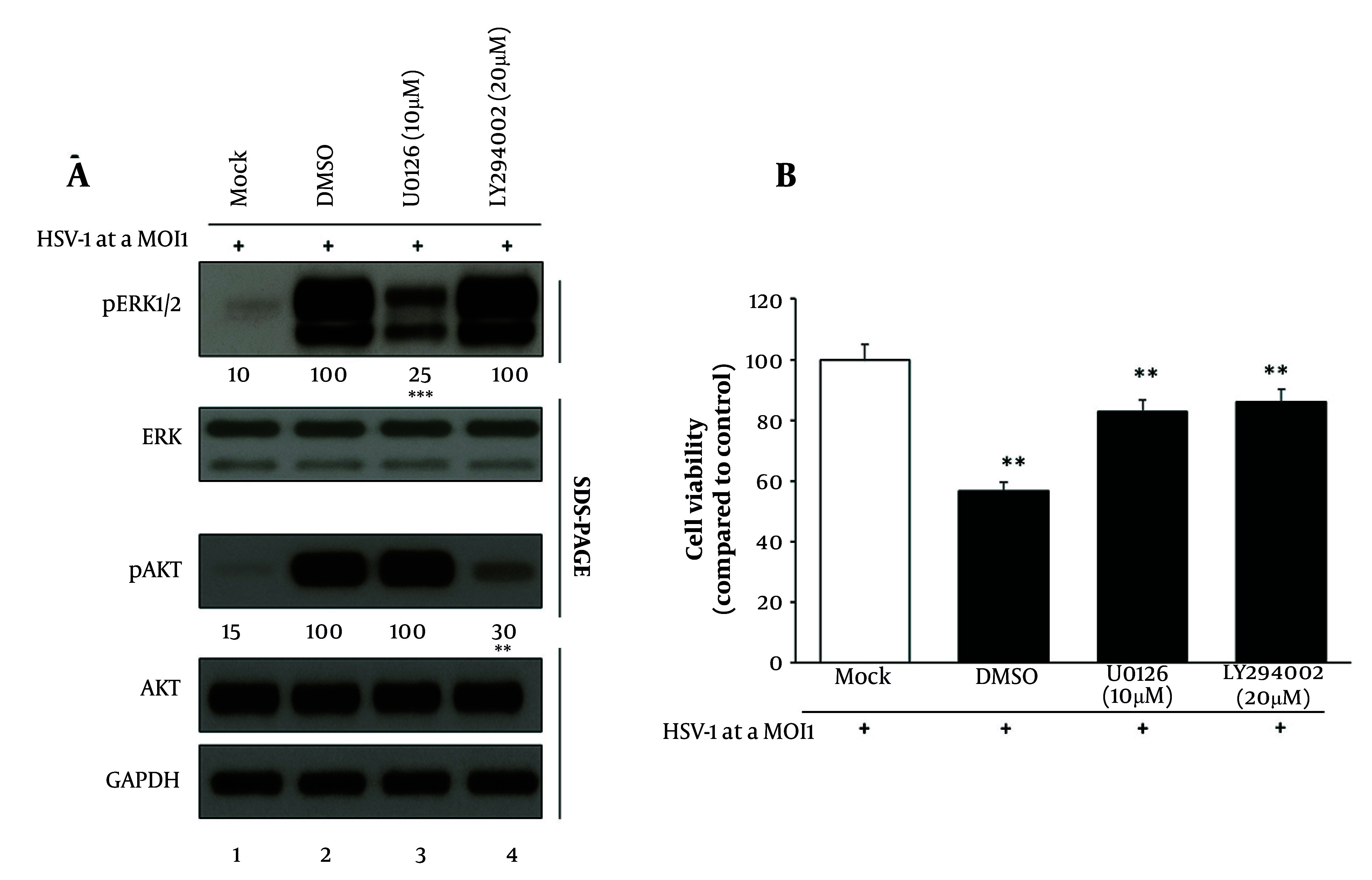
U0126 and LY294002 inhibit extracellular signal-regulated kinase 1/2 (ERK1/2) and phosphatidylinositol 3-kinase/protein kinase B (PI3K/AKT) signaling and restore cell viability in Herpes simplex virus type 1 (HSV-1)-infected HaCaT cells: A, Western blot analysis showing reduced phosphorylated extracellular signal-regulated kinase 1/2 (p-ERK1/2) levels following U0126 treatment and reduced phosphorylated activation of protein kinase B (p-AKT) after LY294002 treatment and densitometric quantification confirming pathway-specific inhibition; B, MTT assay indicating partial recovery of viability in inhibitor-treated cells relative to untreated infection. GAPDH served as the internal control [data are expressed as mean ± standard deviation (SD); n = 3; ** P < 0.01, *** P < 0.001 vs. control].

To determine the functional roles of the MAPK and PI3K/AKT pathways during HSV-1 infection, HaCaT cells were treated with the MEK1/2 inhibitor U0126 or the PI3K inhibitor LY294002, followed by assessment of pathway activity and cell viability. Western blot analysis (Figure 3A) showed that HSV-1 infection alone induced robust phosphorylation of both ERK1/2 and AKT. Treatment with U0126 effectively suppressed ERK1/2 phosphorylation, reducing it to 25%, while leaving AKT phosphorylation unaffected. Conversely, LY294002 specifically decreased p-AKT levels to 30%, with no reduction in p-ERK1/2 levels. Total ERK1/2, total AKT, and GAPDH loading controls remained unchanged across all conditions, confirming pathway-specific inhibition without nonspecific effects.

Corresponding cell viability was assessed using an MTT assay (Figure 3B). The HSV-1 infection led to a marked reduction in metabolic activity, with viability declining to 56% relative to uninfected controls. However, treatment with U0126 and LY294002 partially restored viability, increasing it to 82% and 86%, respectively. These findings indicate that pharmacological inhibition of either signaling pathway mitigates virus-induced cytotoxic effects, likely by disrupting the cellular mechanisms exploited by HSV-1 for propagation and host modulation.

### 4.4. Confocal Microscopy Reveals Distinct Subcellular Localization Patterns of Phosphorylated Extracellular Signal-Regulated Kinase 1/2, Phosphorylated Activation of Protein Kinase B, and Herpes Simplex Virus Type 1 Following Pathway Inhibition

**Figure 4.**
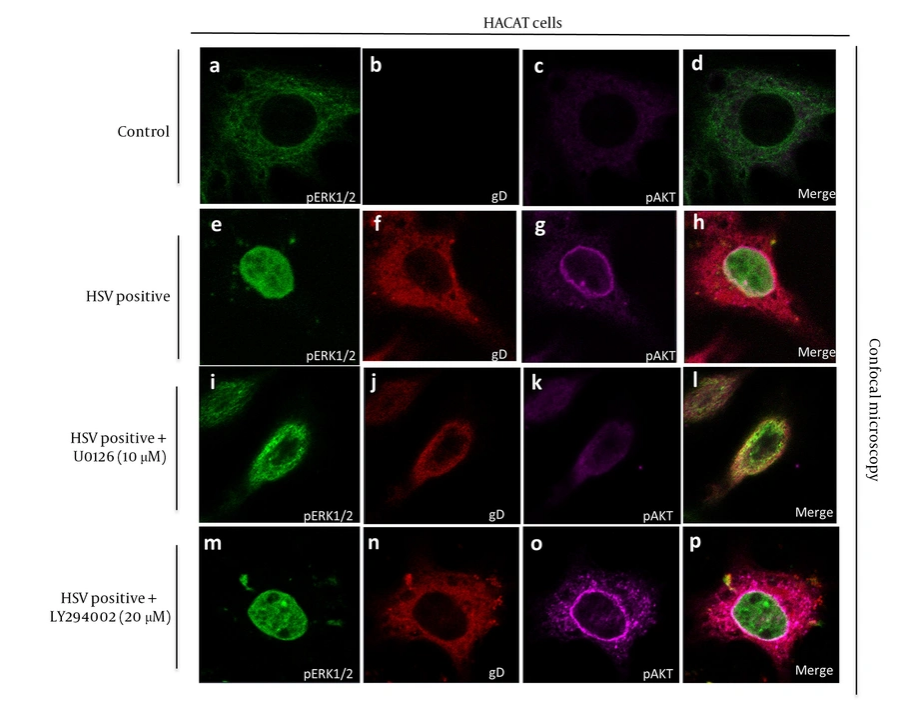
Pathway inhibition alters nuclear localization of extracellular signal-regulated kinase 1/2 (ERK1/2) and activation of protein kinase B (AKT) in herpes simplex virus type 1 (HSV-1)-infected keratinocytes: A - D, confocal images showing nuclear accumulation of phosphorylated extracellular signal-regulated kinase 1/2 (p-ERK1/2) and phosphorylated activation of protein kinase B (p-AKT) in control cells; E - H, confocal images showing nuclear accumulation of p-ERK1/2 and p-AKT in HSV-1-infected cells; I - L, U0126 treatment restricts p-ERK1/2 to the cytoplasm, while LY294002 prevents p-AKT nuclear localization; M - P, HSV-1 glycoprotein D (gD) remains cytoplasmic across conditions, confirming that inhibitors affect post-entry signaling. Representative images from three independent experiments are shown.

To visualize the subcellular dynamics of ERK1/2 and AKT activation during HSV-1 infection and after pharmacological inhibition, confocal immunofluorescence microscopy was performed (Figure 4). The upper panel (mock) showed basal expression of both p-ERK1/2 and p-AKT restricted to the cytoplasm, with no detectable HSV-1 signal (gD), confirming the uninfected state. In HSV-1-infected cells (second panel), strong nuclear localization of p-ERK1/2 was observed, indicating MAPK pathway activation. The p-AKT displayed perinuclear and nuclear accumulation, while HSV-1 gD staining revealed predominant cytoplasmic localization, with occasional perinuclear signal, consistent with active viral infection and replication processes.

Treatment with the MEK1/2 inhibitor U0126 (third panel) resulted in marked reduction of nuclear p-ERK1/2 signal, with staining largely retained in the cytoplasm. The p-AKT distribution remained unaffected, while HSV-1 signal persisted in the cytoplasm, indicating that MAPK inhibition disrupted ERK nuclear translocation without blocking viral entry. In contrast, treatment with the PI3K inhibitor LY294002 (fourth panel) suppressed p-AKT nuclear localization, causing cytoplasmic retention. The p-ERK1/2 remained strongly expressed in both cytoplasmic and nuclear compartments. The HSV-1 gD signal was again evident in the cytoplasm, indicating continued infection despite AKT pathway inhibition.

These spatial patterns support the conclusion that HSV-1 induces compartmentalized activation of ERK and AKT signaling, and that pathway-specific inhibition alters nuclear translocation without eliminating viral presence. This highlights the importance of nuclear signaling events in mediating HSV-1-host interactions.

### 4.5. Pharmacological Inhibition of Extracellular Signal-Regulated Kinase 1/2 and Phosphatidylinositol 3-Kinase/Protein Kinase B Reduces Herpes Simplex Virus Type 1 Replication in HaCaT Cells

**Figure 5.**
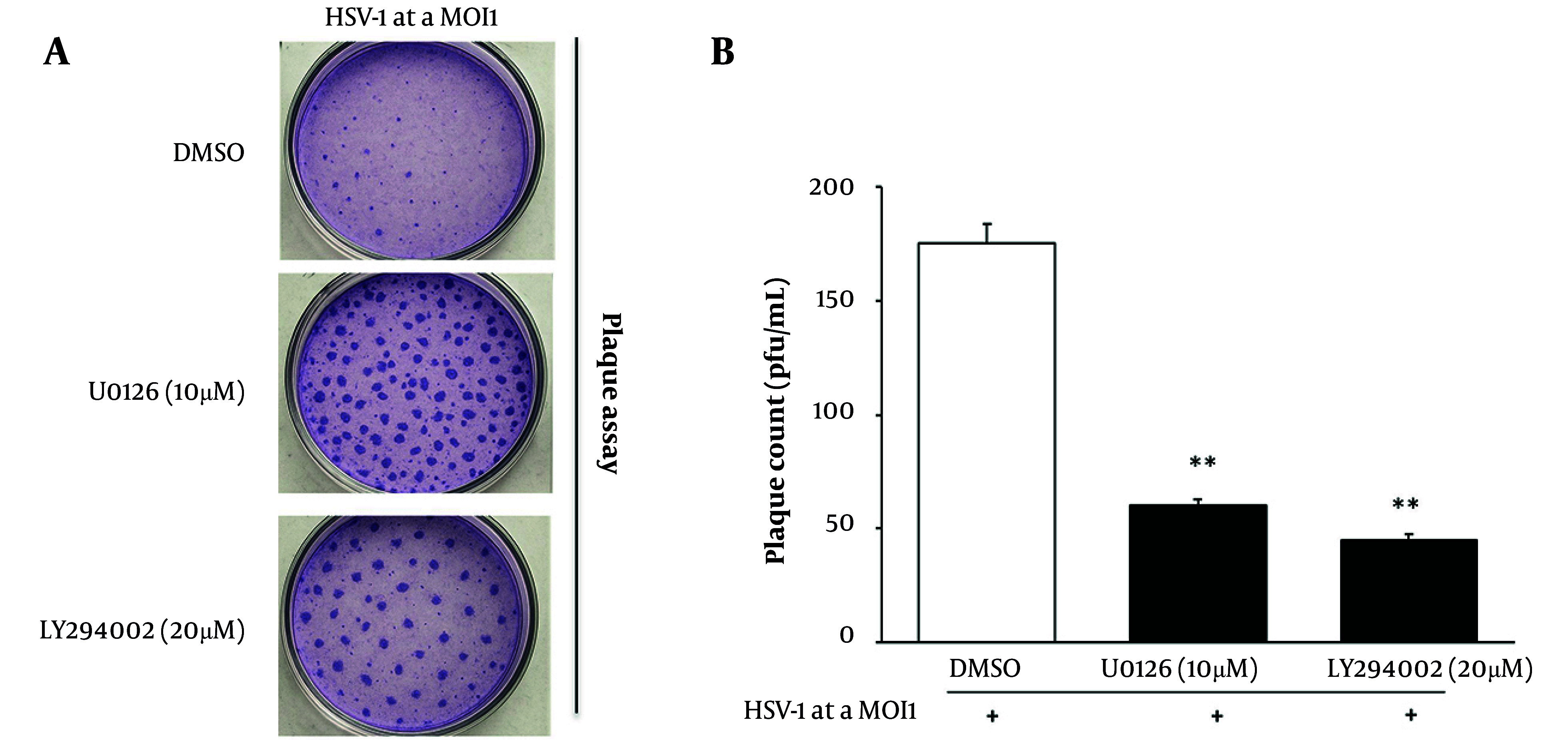
Pharmacological inhibition of extracellular signal-regulated kinase 1/2 (ERK1/2) and phosphatidylinositol 3-kinase/protein kinase B (PI3K/AKT) signaling suppresses herpes simplex virus type 1 (HSV-1) replication: A, plaque formation is markedly reduced in cells treated with U0126 or LY294002 compared with untreated infection; B, quantification of plaque-forming units (PFU) showing significant declines in infectious viral titers in inhibitor-treated groups [data are expressed as mean ± standard deviation (SD); n = 3; ** P < 0.01 vs. control].

To assess the impact of ERK1/2 and PI3K/AKT signaling on HSV-1 replication, a plaque assay was performed in HaCaT cells treated with either U0126 (MEK1/2 inhibitor) or LY294002 (PI3K inhibitor) following viral infection (1 hour pretreatment, inhibitors maintained throughout infection). As shown in Figure 5A, untreated HSV-1-infected wells exhibited extensive plaque formation (~150 - 200 plaques/well), indicating robust viral replication. In contrast, U0126-treated wells showed a marked reduction (~50 - 70 plaques/well), whereas LY294002 treatment further decreased plaque formation to ~30 - 50 plaques/well, reflecting attenuated viral propagation. Quantitative analysis (Figure 5B) confirmed these observations: the infected control averaged ~175 plaques/well, while U0126 and LY294002 treatments significantly reduced counts to ~60 and ~45 plaques/well, respectively (P < 0.01 vs. infected control). These results validate that both ERK1/2 and PI3K/AKT pathways support HSV-1 replication, and that their inhibition substantially restricts productive infection in epithelial cells.

## 5. Discussion

Cancer and infectious diseases present a significant global health burden due to their high prevalence, morbidity, and impact on quality of life. Cancer is a leading cause of death worldwide, with complex pathogenesis involving genetic, environmental, and lifestyle factors. The management of cancer often requires advanced treatment strategies and ongoing research to better understand tumor biology and improve therapeutic outcomes (18, 19). Infectious diseases, including viral infections such as HSV-1, also pose a major health challenge (20-22). The HSV-1 is known for causing recurrent oral infections that significantly affect patients' daily lives. These infections can lead to discomfort, pain, and social embarrassment, emphasizing the need for effective management and prevention strategies (5, 6).

Our study demonstrates that HSV-1 infection robustly activates the mitogen-activated protein kinase/extracellular signal-regulated kinase 1/2 (MAPK/ERK1/2) and PI3K/AKT signaling pathways in HaCaT cells, contributing to enhanced viral gene expression, host inflammatory responses, and progressive loss of cellular viability. These findings align with accumulating evidence that many viruses, including herpesviruses, exploit host intracellular signaling cascades to create a permissive environment for their replication and persistence (23, 24). Consistent with prior reports in HEp-2, Vero, and neuronal models (23, 24), our results indicate that ERK1/2 activation serves a dual function — supporting early viral gene transcription while simultaneously suppressing innate immune signaling. The present data extend these observations to human keratinocytes, the physiologically relevant site of HSV-1 reactivation, highlighting that the same signaling dependency is preserved across cell types.

Similarly, the PI3K/AKT pathway is a well-documented target for viral manipulation. The HSV-1 is known to activate AKT early during infection to suppress apoptosis and prolong host cell survival (25). In our model, AKT phosphorylation was rapidly induced and sustained up to 24 hours. Treatment with LY294002 significantly suppressed p-AKT levels and reduced viral plaque formation, confirming the functional importance of this pathway. This parallels findings from Liu and Cohen (25) and Maddaluno et al. (26), who demonstrated that pharmacological or genetic inhibition of PI3K/AKT curtailed replication of multiple herpesviruses. Our study therefore reinforces PI3K/AKT as a conserved signaling axis exploited by HSV-1 and extends these insights by linking its inhibition to reduced inflammatory gene output in epithelial cells.

Subcellular localization analysis via confocal microscopy further supported our biochemical findings. The nuclear accumulation of p-ERK1/2 and p-AKT observed here mirrors patterns seen during Epstein–Barr and cytomegalovirus infections, suggesting that nuclear translocation is a general viral strategy to modulate host transcriptional machinery. Inhibitor treatment prevented this nuclear translocation, highlighting the importance of subcellular dynamics in signaling control (19).

Our transcriptional data revealed strong upregulation of viral immediate-early (ICP0, ICP4) and late (gB, gC) genes, indicative of active lytic infection. This was accompanied by increased expression of host inflammatory mediators IL-6 and TNF-α. These cytokines are known contributors to HSV-1-induced inflammation and tissue damage (27). Inhibiting ERK1/2 and PI3K/AKT not only suppressed viral replication but also attenuated cytokine gene expression, demonstrating the dual role of these pathways in promoting viral propagation and host inflammation.

A recent review by Lottini et al. emphasized that fibroblast growth factor receptor (FGFR)-ERK signaling drives ocular inflammation in HSV-1 infection and that FGFR inhibition reduces disease severity (28). Recent literature further supports these interpretations. Lobo et al. (29) demonstrated that modulation of oxidative and inflammatory responses improves outcomes in HSV-related keratitis, highlighting the therapeutic potential of targeting host signaling cascades. Similarly, Gomes et al. (30) and Hussain et al. (31) explored host–pathogen interaction and inflammatory biomarker regulation in infectious and inflammatory diseases, providing additional mechanistic parallels to our findings.

Plaque assays provided functional confirmation, showing that both U0126 and LY294002 led to significant reductions in infectious viral output. These findings align with studies showing that blocking ERK/AKT activation using FGFR or Src kinase inhibitors reduces HSV-1 replication (26-28). Although this study focused on single-pathway inhibition, we acknowledge that dual targeting of the ERK1/2 and PI3K/AKT pathways could yield additional insights. Pilot observations indicated that combined treatment with U0126 and LY294002 caused substantial cytotoxicity in uninfected HaCaT cells (> 60% reduction in viability within 12 hours), making it difficult to distinguish antiviral effects from nonspecific toxicity. For this reason, we prioritized dissecting the individual contributions of each pathway. Future studies will investigate optimized combinatorial dosing or transient inhibition strategies to evaluate potential synergistic antiviral effects while minimizing cytotoxicity.

**Figure 6.**
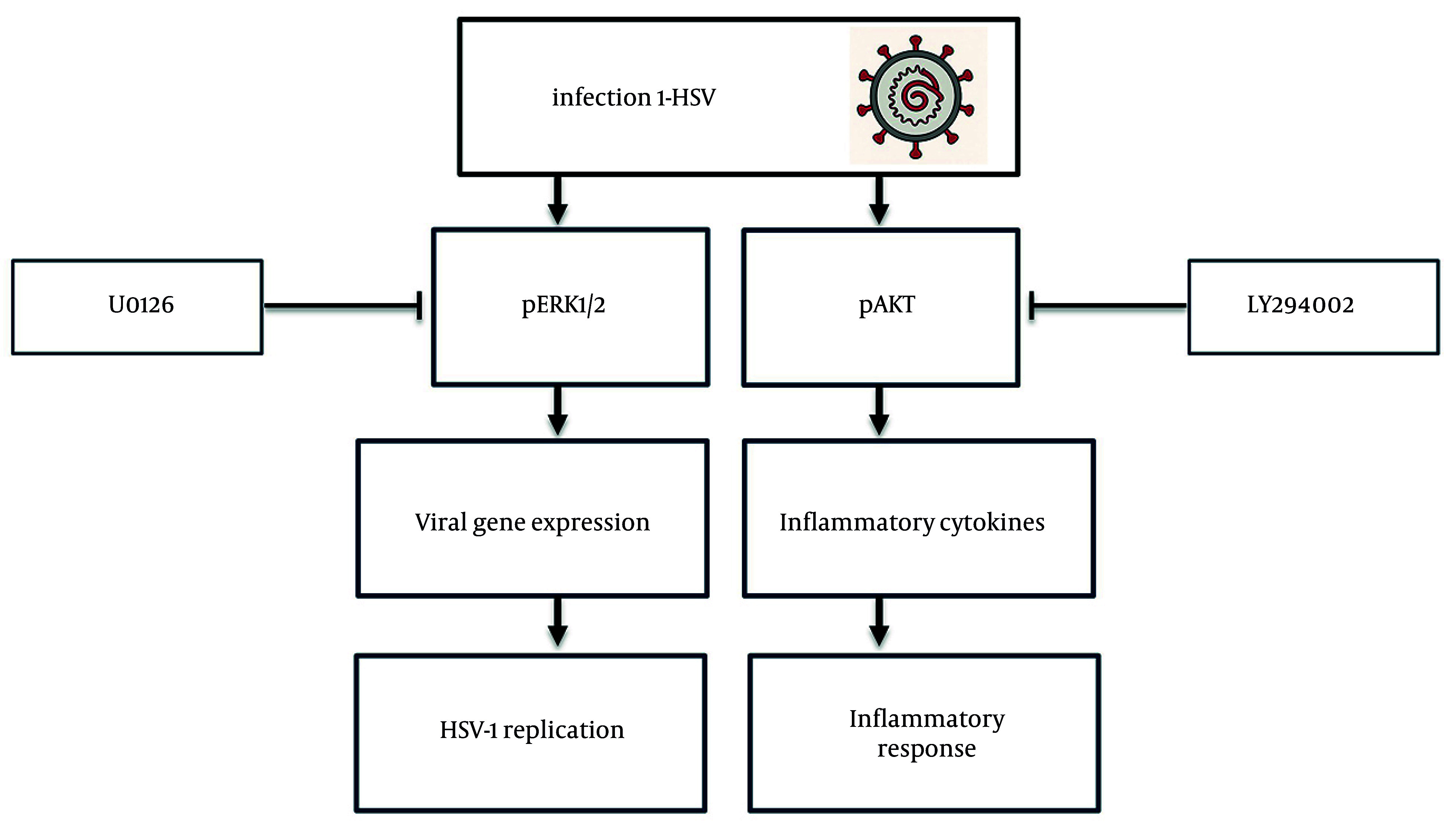
Schematic model summarizing herpes simplex virus type 1 (HSV-1)-induced signaling and the effects of pathway inhibition; HSV-1 activates mitogen-activated protein kinase/extracellular signal-regulated kinase 1/2 (MAPK/ERK1/2) and phosphatidylinositol 3-kinase/protein kinase B (PI3K/AKT) pathways, promoting viral replication and inflammatory gene expression. U0126 and LY294002 disrupt pathway activation, inhibit nuclear translocation of phosphorylated extracellular signal-regulated kinase 1/2 (p-ERK1/2) and phosphorylated activation of protein kinase B (p-AKT), and reduce viral replication and cytokine release.

Collectively, these findings provide a mechanistic model of HSV-1-induced signaling and pharmacological intervention. Upon infection, HSV-1 activates the MAPK and PI3K/AKT pathways, leading to ERK1/2 and AKT phosphorylation and nuclear translocation. These events promote viral gene expression and proinflammatory cytokine induction (IL-6 and TNF-α), resulting in reduced cell viability and increased cytotoxicity. Treatment with U0126 and LY294002 effectively disrupts these processes, decreasing viral replication and restoring cell survival. This integrated schematic (Figure 6) encapsulates the dual role of HSV-1-induced signaling in promoting both viral propagation and host cell damage, underscoring the therapeutic relevance of targeting ERK1/2 and AKT pathways.

Taken together, our data reinforce the concept that HSV-1 co-opts host ERK1/2 and PI3K/AKT pathways to promote viral gene expression, evade host defenses, and induce inflammation. Targeting these host pathways offers a promising strategy for controlling HSV-1 infections, especially in the context of drug resistance or recurrent disease. This study elucidates the critical role of ERK1/2 and PI3K/AKT signaling in HSV-1–infected keratinocytes. We demonstrate that HSV-1 induces sustained phosphorylation and nuclear translocation of ERK1/2 and AKT, which correlates with increased viral gene expression, cytokine production, and loss of cell viability. Pharmacological inhibition of these pathways significantly suppressed viral replication and restored cell survival, underscoring the therapeutic potential of host-directed antiviral approaches.

### 5.1. Conclusions

This study provides critical insights into how HSV-1 infection modulates host cell signaling pathways to facilitate viral replication and induce inflammatory responses in human keratinocytes. We demonstrate that HSV-1 activates both the ERK1/2 and PI3K/AKT pathways in a time-dependent manner, leading to increased viral gene expression, cytokine production, and reduced cell viability. Pharmacological inhibition of these pathways significantly suppressed viral replication, restored cell survival, and reduced pro-inflammatory gene expression. These findings highlights that by curbing orolabial HSV-1 replication through MEK/ERK and PI3K/AKT co-targeting, this approach may reduce recurrent lesions and help preserve oral mucosal integrity and overall oral health.

## Data Availability

The dataset presented in the study is available on request from the corresponding author during submission or after publication.
